# Supplementary material for: WordCommentsAnalyzer: A windows software tool for qualitative research
Source: F1000Res. 2018 Sep 4;7:536. Originally published 2018 May 3. [Version 2] doi: 10.12688/f1000research.14819.2 (PMC6137411; doi:10.12688/f1000research.14819.2)
Supplement: Supplementary file 3 [file f1000research-7-17614-s0002.tgz › b2a79496-4ada-47c4-9682-a99ce867836d.zip!/Eight Iranian Health Journals/Salmand_Iranian Journal of Ageing _2.docx]

© Salmand_Iranian Journal of Ageing . This work is licensed under CC BY-NC 4.0 (https://creativecommons.org/licenses/by-nc/4.0/).

Volume & Issue: دوره 12 - شماره 4

Title: اثربخشی فعالیت جسمانی بر انعطاف‌پذیری شناختی و کمال‌گرایی سالمندان

http://salmandj.uswr.ac.ir/article-1-1190-fa.html

اهداف با توجه به کاهش فعالیت جسمانی و اثرگذاری آن بر ویژگی‌های روان‌شناختی در سالمندان، تحقیق حاضر با هدف اثربخشی سطح فعالیت جسمانی بر انعطاف‌پذیری شناختی و کمال‌گرایی سالمندان انجام شد. مواد و روش ها روش تحقیق حاضر از نوع علّی مقایسه‌ای است. جامعه آماری را سالمندان مناطق یک و سه تهران که در سرای محله، کانون سالمندان، پارک‌ها، بوستان‌ها و مناطق تفریحی حضور داشتند، تشکیل داد. آزمودنی‌ها شامل ۱۲۶ سالمند از استان تهران بودند که با روش نمونه‌گیری مبتنی بر هدف انتخاب شدند. سالمندان پرسش‌نامه‌های جمعیت‌شناختی، بررسی وضعیت شناختی سالمندان، سطح فعالیت جسمانی چامپس استوارت (۲۰۰۱)، کمال‌گرایی هیل (۲۰۰۴) و انعطاف‌پذیری شناختی دنیس و وندروال (۲۰۱۰) را کامل کردند. برای تحلیل یافته ها از تحلیل واریانس یک‌راهه، ضریب همبستگی پیرسون و رگرسیون چندمتغیره استفاده شد. یافته ها نتایج نشان داد بین انعطاف‌پذیری شناختی و کمال‌گرایی در سه گروه سالمندان با فعالیت جسمانی کم، متوسط و زیاد تفاوت از نظر آماری معنی‌دار است و سالمندان با فعالیت بدنی زیاد انعطاف‌پذیری شناختی و کمال‌گرایی بهتری دارند (۰/۰۵>P). بین سطح فعالیت جسمانی با کمال گرایی و انعطاف‌پذیری شناختی رابطه وجود دارد. همچنین متغیرهای ادارک کنترل و ادراک توجیهات، متغیر کمال‌گرایی را در سالمندان پیش‌بینی می‌کند (۰/۰۵>P). نتیجه گیری افراد سالمند با انجام فعالیت جسمانی می‌توانند انعطاف‌پذیری شناختی و کمال‌گرایی خود را بهبود بخشند. بنابراین انتخاب سبک زندگی فعال به منظور ارتقای عوامل شناختی توصیه می‌شود.

Title: طراحی و روان‌سنجی ابزار اندازه‌گیری سالمندی فعال ایرانی

http://salmandj.uswr.ac.ir/article-1-1287-fa.html

اهداف پژوهش حاضر با هدف طراحی و تعیین ویژگی‌های روان‌سنجی ابزار اندازه‌گیری سالمندی فعال متناسب با معیارهای فرهنگی جامعه سالمندی ایرانی انجام شد. مواد و روش ها این مطالعه پژوهشی روش‌شناختی و بخشی از مطالعه بزرگ‌تر با عنوان «فهم سالمندی فعال و ساخت ابزار اندازه‌گیری آن» با روش تحقیق ترکیبی بود که اینگرسلدایتون (۲۰۱۱)، در سال ۱۳۹۴-۱۳۹۵ در دو مرحله با به‌کارگیری گام‌های مطرح‌شده آن را انجام داد. در مرحله اول با استفاده از مطالعه کیفی و پیشینه مطالعاتی در سه گام ابزار اندازه‌گیری اولیه طراحی شد. در مرحله دوم طی دو گام شامل: ۱. بررسی اعتبار صوری و محتوا در گروه سالمندان و متخصصان ۲. انجام بررسی پیمایشی و آزمون روان‌سنجی، ویژگی‌های روان‌سنجی ابزار بررسی شد. با روش نمونه‌گیری خوشه‌ای سه‌مرحله‌ای در نمونه‌ای از ۳۵۰ سالمند مراجعه‌کننده به خانه‌های سلامت شهرداری تهران، بررسی انجام شد. نمونه پژوهش شامل سالمندان ۶۰ سال و بالاتر با میانگین سنی ۶۹ سال (SD:۶/۷۸) بود و ۵۲ درصد نمونه، زن (n=۱۸۲) و ۴۸ درصد آن، مرد (n=۱۶۸) بود. از نرم‌افزار ۱۶ SPSS برای اجرای آزمون‌های آماری شامل تحلیل عاملی اکتشافی، تحلیل گویه، ضریب آلفای کرونباخ و ضریب همبستگی پیرسون استفاده شد. یافته ها نتیجه مرحله اول عبارت بود از: ابزار اولیه‌ای با ۶۱ سؤال با طیف پاسخ لیکرتی پنج‌درجه‌ای (هیچ تا خیلی زیاد). در مرحله دوم در بررسی اعتبار صوری و محتوای ابزار، شش سؤال حذف و ابزار اندازه‌گیری ۵۵ سؤالی حاصل شد. نتایج آزمون روان‌سنجی ابزار، پس از تحلیل گویه‌ها و تحلیل عاملی اکتشافی به ابزار اندازه‌گیری نهایی ۴۰ سؤالی با شش عامل جماعت‌پذیری (مشارکت اجتماعی‌نهادی)، ذهن‌ورزی، تعامل‌گرایی، بینش عاملیت‌گرا (فعال)، نقش‌آفرینی و پویایی جسمی‌عملکردی با قابلیت تبیین ۵۶ درصد کل واریانس سالمندی فعال منجر شد. پایایی مبتنی بر محاسبه ضریب آلفای کرونباخ، مؤید همسانی درونی مطلوب پرسش‌نامه ۰/۸۸ و ثبات از طریق بازآزمایی ۰/۹۵ بود (P<۰/۰۱). نتیجه گیری ابزار اندازه‌گیری سالمندی فعال حاضر که متناسب با جامعه سالمندی ایرانی است، ضمن تأیید وجود ابعاد ذهنی و رفتاری سالمندی فعال با ۴۰ سؤال و شش عامل، اعتبار و پایایی مطلوبی دارد.

Title: شناسایی عوامل تسهیلگر و بازدارنده کیفیت زندگی در زنان و مردان سالمند (تحقیق پدیدارشناسی)

http://salmandj.uswr.ac.ir/article-1-1248-fa.html

اهداف با توجه به رشد سریع جمعیت سالمندان در جهان و به ویژه در ایران، ضرورت بررسی کیفیت زندگی سالمندان آشکار می‌شود. از این رو شناسایی عوامل مؤثر بر مسائل سالمندی اهمیت خاصی دارد. هدف پژوهش حاضر شناسایی عوامل تسهیلگر و بازدارنده کیفیت زندگی در زنان و مردان سالمند است. مواد و روش ها پژوهش حاضر مطالعه‌ای کیفی و از نوع پدیدارشناختی است. شرکت‌کنندگان در این تحقیق ۲۳ نفر از سالمندان بودند که به روش نمونه‌گیری هدفمند انتخاب شدند. این شرکت‌کنندگان از ۶۱ تا ۱۰۱ ساله بودند که ۶۰/۹ درصد آن‌ها را زنان و ۳۹/۱ درصدشان را مردان تشکیل می‌دادند. داده‌ها از طریق مصاحبه‌های عمیق و نیمه‌ساختاریافته جمع‌آوری شدند. مصاحبه‌ها ضبط شدند و به روش استرابرت و کارپنتر تجزیه‌وتحلیل شدند. یافته ها یافته‌های پژوهش شش مضمون اصلی را شامل می‌شود که عبارتند از: بسترهای تسهیل‌گرایانه داخلی، عوامل خارجی پیش‌برنده، چالش‌ها و مشکلات درون‌فردی، موانع و کمبودهایی در عرصه برونی، خواسته‌ها و آرزوهای زوال رفته، بایسته‌ها و پیشنهاداتی برای بهبود. همچنین ۲۴ مضمون فرعی دارد. نتیجه گیری طبق نتایج این پژوهش برای بهبود زندگی سالمندان باید به عواملی نظیر نقش باورهای مذهبی، تغذیه مناسب، ملاقات اقوام، خاطره‌گویی و ایجاد روابط اجتماعی مناسب توجه کرد. همچنین با رفع موانعی از جمله شکستن تابوی ازدواج سالمندی، حمایت مالی و ارائه خدمات مشاوره‌ای برای تأمین سلامت سالمندان و ارتقای کیفیت زندگی‌شان اقدام کرد.

Title: رابطه سبک‌های دلبستگی به خدا و تاب‌آوری با اضطراب مرگ در سالمندان

http://salmandj.uswr.ac.ir/article-1-1296-fa.html

اهداف در جوامع امروزی سالمندان یکی از گروه‌های اجتماعی روبه‌رشد هستند که با توجه به هرم سنی اهمیت روزافزونی یافته‌اند. این مطالعه با هدف بررسی رابطه ساده و چندگانه سبک‌های دلبستگی به خدا و تاب‌آوری با اضطراب مرگ در سالمندان شهر زنجان انجام شده است. مواد و روش ها مطالعه حاضر مطالعه‌ای توصیفی از نوع همبستگی بود. جامعه آماری این پژوهش شامل همه سالمندان شهر زنجان در سال‌های ۹۶-۱۳۹۵ بود که بیش از ۶۵ سال داشتند. به شیوه نمونه‌گیری در دسترس ۲۴۲ نفر انتخاب شدند و برای جمع‌آوری اطلاعات به آن‌ها مراجعه شد. از این تعداد به دلیل همکاری کامل نکردن و مخدوش‌بودن، پرسش‌نامه  ۱۱ نفر کنار گذاشته شد و داده های ۲۳۱ نفر وارد تحلیل شد. ابزار استفاده‌شده در این پژوهش مقیاس اضطراب مرگ تمپلر، پرسش‌نامه  دلبستگی به خدا بک و مک دونالد و مقیاس تاب‌آوری کونور و دیویدسون بود. برای تحلیل داده‌ها از روش‌های آماری ضریب همبستگی پیرسون و تحلیل رگرسیون چندگانه استفاده شد. یافته ها نتایج ضریب همبستگی نشان داد که همبستگی بین اضطراب مرگ با توجه به خدا ۰/۵۶-، با اعتماد در مقابل بی‌اعتمادی ۰/۶۵-، با توکل به خدا ۰/۵۳-، با ارتباط با خدا ۰/۶۸- و بین کل دلبستگی به خدا و اضطراب مرگ ۰/۶۸-، و در نهایت بین تاب‌آوری و اضطراب مرگ ۰/۵۴- به دست آمد که همگی در سطح ۰/۰۱ معنی‌دار هستند. همچنین تحلیل رگرسیون چندگانه نشان داد که متغیرهای پیش‌بین ۶۴ درصد واریانس اضطراب مرگ را تبیین می‌کنند. نتیجه گیری نتایج نشان داد که بین سبک‌های دلبستگی به خدا و تاب‌آوری با اضطراب مرگ رابطه منفی و معنی‌داری وجود دارد. بنابراین توصیه می‌شود مسئولان مربوطه به آموزش و مهارت‌های لازم در خصوص تاب‌آوری و اعتقاد به خدا و خداباوری در دوره سالمندی و مراکز نگهداری سالمندان توجه کنند.

Title: ارتباط ویژگی‌های شخصیتی (روان‌رنجورخویی و برون‌گرایی) و خودکارآمدی با افسردگی سالمندی

http://salmandj.uswr.ac.ir/article-1-1306-fa.html

اهداف پژوهش حاضر ارتباط ویژگی‌های شخصیتی (روان‌رنجورخویی و برون‌گرایی) و خودکارآمدی را با افسردگی دوران سالمندی در معلمان مرد بازنشسته شهر هرسین بررسی می‌کند. مواد و روش ها این پژوهش نوعی مطالعه توصیفی‌همبستگی بود که به صورت مقطعی در سال ۱۳۹۵ انجام شد. شرکت‌کنندگان پژوهش شامل ۱۷۳ نفر از معلمان مرد بازنشسته بودند که با روش نمونه‌گیری تصادفی انتخاب شدند. ابزار جمع‌آوری داده‌ها شامل مقیاس افسردگی سالمندان (GDS)، پرسش‌نامه شخصیتی نئو  (NEO-FFI) و پرسش‌نامه خودکارآمدی عمومی (GSE) بود. یافته ها داده‌های حاصل از اجرای پرسش‌نامه‌ها با استفاده از ضریب همبستگی پیرسون و رگرسیون برای بررسی نقش پیش‌بینی‌کننده ویژگی‌های شخصیتی (روان‌رنجورخویی و برون‌گرایی) و خودکارآمدی در پیش‌بینی افسردگی دوران سالمندی تحلیل شدند. نتایج نشان داد که بین افسردگی دوران سالمندی با برون‌گرایی (r=۰/۶۵، P<۰/۰۰۱) و خودکارآمدی (r=۰/۶۶، P<۰/۰۰۱) رابطه منفی معنی‌داری و بین روان‌رنجورخویی و افسردگی دوران سالمندی رابطه مثبت معنی‌داری (r=۰/۵۴، P<۰/۰۰۱) وجود دارد. همچنین یافته‌های حاصل از رگرسیون نشان داد که متغیرهای مستقل در مجموع ۵۰ درصد افسردگی دوران سالمندی را پیش‌بینی می‌کنند. نتیجه گیری بر اساس نتایج می‌توان بیان کرد که روان‌رنجورخویی می‌تواند باعث افزایش و برون‌گرایی و خودکارآمدی موجب کاهش افسردگی دوران سالمندی شود.

Title: رابطه بین خودتنظیمی با اعتماد به تعادل در سالمندان مرد فعال و غیر فعال

http://salmandj.uswr.ac.ir/article-1-1313-fa.html

اهداف هدف پژوهش حاضر بررسی ارتباط بین خودتنظیمی با اعتماد به تعادل در سالمندان فعال و غیر فعال شهرستان مهاباد بود.  مواد و روش ها روش تحقیق از نظر هدف کاربردی و از نظر شیوه گردآوری اطلاعات مقطعی‌همبستگی بوده است. جامعه آماری این تحقیق را تمامی سالمندان ۶۰ تا ۸۵ سال شهرستان مهاباد تشکیل دادند. ۹۳ سالمند فعال و ۹۳ سالمند غیرفعال به عنوان نمونه به صورت تصادفی در دسترس در نظر گرفته شدند. ابزار گردآوری داده‌ها پرسش‌نامه اعتماد به تعادل (ABC) و پرسش‌نامه خودتنظیمی (SRI-۲۵) بود. تجزیه‌وتحلیل داده‌ها با استفاده از آزمون‌های کلموگروف اسمیرنوف، ضریب همبستگی پیرسون، تحلیل رگرسیون چندگانه هم‌زمان، تی مستقل انجام شده است. این پژوهش با استفاده از نرم‌فزار SPSS انجام شد.  یافته ها نتایج مشخص کرد تمامی ابعاد خودتنظیمی با اعتماد به تعادل در سالمندان رابطه مثبت و معناداری داشتند (P≤۰/۰۵).به جز مؤلفه آشکارسازی احساسات (P=۰/۲۵۰) و نیازها، دیگر ابعاد خودتنظیمی می‌توانند بر اعتماد به تعادل تأثیر بگذارند. همچنین مشخص شد سالمندان فعال (اعتماد به تعادل: ۳/۲۴۴۶±۰/۶۳۷۳۵؛ خودتنظیمی: ۳/۸۰۱۷±۰/۷۵۹۴۳) و غیرفعال (اعتماد به تعادل: ۲/۳۴۶۸±۰/۶۹۴۵۴؛ خودتنظیمی: ۲/۸۲۰۸±۰/۷۹۷۸۲) تفاوت معناداری داشتند (P≤۰/۰۵). نتیجه گیری یافته‌های این پژوهش تأیید می‌کند که عدم‌خودتنظیمی در سالمندان می‌تواند به محدودیت و کاهش فعالیت جسمانی در آن‌ها منجر شود. پیشنهاد می‌شود به خودتنظیمی در سالمندان تأکید شود تا خودشان بتوانند بر مشکلات محیطی چیره شوند و توانایی اعتماد به تعادل در آن‌ها ارتقا یابد.

Title: اثربخشی معنادرمانی گروهی بر امید به زندگی و بهزیستی روانی و اجتماعی سالمندان زن ساکن آسایشگاه‌های شهر دبی

http://salmandj.uswr.ac.ir/article-1-1319-fa.html

اهداف هدف پژوهش حاضر، بررسی اثربخشی معنادرمانی گروهی بر امید به زندگی و بهزیستی روانی و اجتماعی سالمندان زن ساکن خانه سالمندان شهر دبی بود. مواد و روش ها این پژوهش نیمه‌آزمایشی از نوع پیش‌آزمون پس‌آزمون همراه با گروه شاهد بود. جامعه آماری پژوهش شامل همه سالمندان زن بیشتر از ۶۵ سال ساکن آسایشگاه‌های شهر دبی بود. تعداد ۸۰ نفر به روش نمونه‌گیری تصادفی طبقه‌بندی‌شده انتخاب شدند و در دو گروه آزمایش و شاهد جای گرفتند. گروه آزمایش، معنادرمانی را به صورت گروهی در هشت جلسه و هر جلسه به‌مدت یک ساعت‌ونیم دریافت کرد و گروه شاهد در فهرست انتظار قرار گرفت. شرکت‌کنندگان در این پژوهش، پرسش‌نامه‌امید اشنایدر، پرسش‌نامه‌بهزیستی روان‌شناختی ریف و پرسش‌نامه بهزیستی اجتماعی را در پیش‌آزمون و پس‌آزمون تکمیل کردند. داده‌ها با استفاده از تحلیل کوواریانس چندمتغیره تجزیه‌وتحلیل شدند. یافته ها میانگین و انحراف استاندارد سن شرکت‌کنندگان در گروه آزمایش و شاهد به ترتیب ۱۲/۱۰±۶۵/۱۰ و ۶۷/۱۲±۱۱/۸۰ بود. همچنین، میانگین و انحراف استاندارد نمرات کل متغیرهای بهزیستی روانی (۴۴۵/۳۱±۳۵/۹۰)، امید به زندگی (۳۷/۰۱±۲/۱۳) و بهزیستی اجتماعی (۲۷/۹۹±۳/۰۶) گروه آزمایش، در پس‌آزمون محاسبه شد. نتایج نشان دادند که معنادرمانی گروهی توانسته است باعث افزایش بهزیستی روانی، بهبود مؤلفه‌های امید به زندگی شامل تفکر رهیاب و تفکر پایورانه و همچنین بهبود مؤلفه‌های بهزیستی اجتماعی شامل انسجام اجتماعی، مشارکت اجتماعی، شکوفایی اجتماعی، پیوستگی اجتماعی و پذیرش اجتماعی در زنان سالمند ساکن آسایشگاه‌های شهر دبی شود (۰/۰۰۱>P). نتیجه گیری متخصصان سلامت روان می‌توانند از معنادرمانی گروهی به مثابه راهکاری مؤثر برای بهبود امید به زندگی و بهزیستی روانی‌اجتماعی زنان سالمند ساکن خانه‌های سالمندان استفاده کنند. همچنین پیشنهاد می‌شود تحقیقات آینده، اثربخشی معنادرمانی گروهی دیگر سازه‌های روان‌شناسی مثبت‌گرا بر سالمندان زن و مرد ساکن خانه‌های سالمندان را بررسی کنند.

Title: ارتباط سلامت عمومی با مشخصات جمعیت شناختی مراقبین خانوادگی بازماندگان سکته مغزی

http://salmandj.uswr.ac.ir/article-1-1334-fa.html

اهداف مراقبت از بازماندگان سکته مغزی به علت تقاضای بیش از اندازه بازماندگان، می‌تواند به ایجاد یا تشدید مشکلات جسمی و روانی متعددی برای مراقبان خانوادگی منجر شود. هدف از پژوهش حاضر، تعیین سلامت عمومی مراقبان بازماندگان سکته مغزی و رابطه آن با مشخصات جمعیت‌شناختی بوده است.  مواد و روش ها در این مطالعه مقطعی به روش نمونه‌گیری دردسترس، ۶۰ نفر از بازماندگان سکته مغزی دو مرکز درمانی سینا و فیروزگر استان تهران در سال ۱۳۹۴ بررسی شدند. گردآوری داده‌ها با استفاده از پرسش‌نامه‌های جمعیت‌شناختی و سلامت عمومی گولدبرگ (GHQ) صورت گرفت. برای تجزیه‌و‌تحلیل داده‌ها از آمار توصیفی و استنباطی شامل میانگین انحراف معیار در ۱۵ SPSS V استفاده شد.  یافته ها میانگین نمره کلی به‌دست‌آمده از سلامت عمومی مراقبان ۶۱/۴۱±۱۴/۹ بود که در زنان ۶۳/۰۷±۰۱۶/۰۹ و در مردان ۵۸/۳۳±۱۲/۲۸محاسبه شد. بین نمره کلی سلامت عمومی با وضعیت شغلی و طبقه سنی و وضعیت مراقبتی رابطه معنی‌داری وجود دارد (۰/۰۱=P). مراقبان بیکار، افزایش سن و مراقبت شبانه‌روزی از بازمانده سکته مغزی با کاهش میزان سلامت عمومی ارتباط وجود دارد، ولی بین جنسیت (۰/۳۵=P)، مدت‌زمان مراقبت (۰/۲۴=P)، وضعیت تأهل (۰/۷۱=P) و وضعیت تحصیلی (۰/۰۵=P) با میزان سلامت عمومیِ مراقبان سکته مغزی ارتباط معنی‌داری وجود ندارد.  نتیجه گیری این مطالعه نشان داد مراقبان خانوادگی بازماندگان سکته مغزی، در ابعاد مختلف، کاهش میزان سلامت عمومی را تجربه می‌کنند؛ بنابراین توصیه می‌شود برنامه‌ریزی لازم برای افزایش سلامت عمومی و حمایت اجتماعی لازم از مراقبان خانوادگی، از طریق سیاست‌گذاران حوزه سلامت و درمان انجام شود.

Title: مناسب‌سازی شاخص‌های انتخاب مکان بهینه ورزشی سالمندان با استفاده از مدل تحلیل سلسله مراتبی(AHP) و GIS

http://salmandj.uswr.ac.ir/article-1-1348-fa.html

اهداف امروزه دوران سالمندی و مشکلات آن یکی از چالش‌های موجود در کشور است. این مسئله ضرورت برنامه‌ریزی صحیح برای این دوره را یادآور می‌شود. دولت‌ها در مجموعه سیاست‌های رفاهی خود، فرصت برابر را به عنوان حق مردم بیان می‌کنند و به نمایندگی جامعه در پی تحقق این حق برای عموم مردم هستند. در این راستا، تحقیق حاضر با هدف مناسب‌سازی شاخص‌های انتخاب مکان بهینه ورزشی سالمندان انجام شده است.  مواد و روش ها پژوهش حاضر از نوع توصیفی‌تحلیلی است که با استفاده از ابزار مصاحبه ساختاریافته با هشت نفر از خبرگان به شیوه AHP به وزن‌دهی معیارهای تحقیق پرداخته است. در نتیجه این کار به سه شاخص مهم‌تر در انتخاب امکان ورزشی دست یافته است. در ادامه استخرهای محدوده شهری کرج (27 مورد) به عنوان مورد مطالعه و مشتریان سالمند آن‌ها به عنوان جامعه تحقیق انتخاب شدند که از این میان 127 نفر به شیوه نمونه‌گیری طبقه‌ای بررسی شدند.  پس از وزن‌دهی به زیرمعیارهای مربوط به معیارهای تحقیق، به‌منظور تشکیل پایگاه داده فضایی از نرم‌افزارهای Auto Cad، Excel، ArcGIS، Google Earth استفاده شد. یافته ها سه شاخص دسترسی، مجاورت با کاربری‌های دیگر شهری و ایمنی از دیدگاه خبرگان رتبه بهتری در زمینه تأثیرگذاری بر انتخاب اماکن ورزشی کسب کردند. زیرمعیارهای دسترسی به خطوط مترو و بزرگ‌راه‌ها از معیار دسترسی، مجاورت با پارک‌ها و فضاهای سبز و مجاورت با مراکز فرهنگی از شاخص مجاورت و موقعیت مناسب نسبت به مراکز درمانی و مراکز آتش‌نشانی از شاخص ایمنی، اهمیت نسبی بیشتری در مقایسه با زیرمعیارهای دیگر داشته‌اند.  نتیجه گیری در این پژوهش 127 نفر از سالمندان به عنوان گروه مهمی از مشتریان اماکن ورزشی بررسی شدند. از این تعداد 66 درصد مرد و 34 درصد زن بودند. تقریباً نیمی از نمونه آماری (48 درصد) 60 تا 65 سال داشتند و 54 درصد از آنان بیشتر از شش ساعت در هفته را صرف فعالیت ورزشی می‌کردند. مطابق نتایج، شاخص‌های دسترسی، مجاورت با کاربری‌های دیگر شهری و ایمنی مهم شناخته‌شده‌اند. با استفاده از نتایج این‌گونه تحقیقات و از طریق مکان‌یابی صحیح اماکن ورزشی می‌توان از بروز اشتباه در انتخاب محل احداث اماکن ورزشی جلوگیری کرد.

Title: شناسایی برخی عوامل خطر زمان تا مرگ سالمندان، با به‌کارگیری مدل آمیخته نیمه‌پارامتری مخاطرات رقیب تحلیل بقا

http://salmandj.uswr.ac.ir/article-1-1382-fa.html

اهداف  با توجه به افزایش جمعیت سالمندان ایران، آشنایی با علل مختلف در مرگ سالمندان ضروری است. هدف این مطالعه شناسایی عوامل خطر زمان بقای سالمندان با به‌کارگیری مدل آمیخته نیمه‌پارامتری مخاطرات رقیب بوده است. مواد و روش ها این مطالعه، از نوع هم‌گروهی تاریخی بود. داده‌های مربوط به پرونده ۵۱۰ سالمند بیشتر از ۶۰ سال که از اوایل سال ۱۳۷۹ تا اواخر سال ۱۳۹۱ در سرای سالمند گلابچی کاشان پذیرش‌شده بودند، تمام شماری و مطالعه شد. به‌منظور بررسی و شناسایی برخی عوامل خطر مربوط به زمان تا مرگ سالمندان، مدل آمیخته نیمه پارامتری در حضور مخاطرات رقیب تحلیل بقا، به داده‌ها برازش شد. مرگ ناشی از بیماری‌های قلبی‌عروقی به‌عنوان حادثه مهم مدنظر و دیگر علل مرگ سالمندان به‌ عنوان مخاطره رقیب در مدل در نظر گرفته شده‌اند. برای برآورد پارامترهای مدل، الگوریتم EMC به‌کار رفت و پارامترها و حدود اطمینان آن‌ها با احتمال ۹۵ درصد، در نرم‌افزار R ۳.۳.۱ برآورد شد. یافته ها در این داده‌ها مرگ به علل قلبی در سالمندان ۱۹ درصد و مرگ به دیگر علل ۴۴ درصد بود. ۳۷ درصد نیز به عنوان داده سانسور شده بود. در مدل‌های برازش شده تک‌متغیره سن و وضعیت حرکتی بر زمان تا مرگ سالمندان دچار بیماری‌های قلبی‌عروقی اثر معنادار داشتند. همچنین عوامل سن ((۳/۴۲, ۱/۳۵)=Cl); (HR=۳/۲۲))، مشکل کلیوی، فشار خون بالا، سابقه سکته مغزی و سابقه سکته قلبی بر زمان تا مرگ سالمندان دچار مخاطره رقیب معنادار شدند. در برازش مدل چندمتغیره، سن بر زمان تا مرگ سالمندان دچار بیماری‌های قلبی‌عروقی دارای اثر معنادار شد. همچنین عوامل سن، مشکل کلیوی، چربی خون بالا و میزان تحرک بر زمان تا مرگ سالمندان دچار بیماری‌های غیر قلبی‌عروقی به عنوان مخاطره رقیب معنادار شدند.   نتیجه گیری در برازش‌های تک‌متغیره عوامل سن، سابقه سکته قلبی، سابقه سکته مغزی و مشکلات کلیوی به عنوان عامل خطر بر زمان تا وقوع مرگ سالمندان شناسایی شدند. همچنین در برازش چندمتغیره عامل سن بر زمان تا مرگ با علل قلبی و عوامل مشکلات کلیوی، سن، وضعیت تحرک و چربی خون بالا بر زمان تا مرگ سالمندان دچار علل غیر قلبی شناسایی شدند.
